# Analysis of the surgical treatment of fracture in HIV positive patients: A clinical study

**DOI:** 10.12669/pjms.336.13368

**Published:** 2017

**Authors:** Guangyong Xu, Juan Zhao, Jie Sun, Yizong Liu

**Affiliations:** 1 Guangyong Xu, Department of Dermatology, Qingdao No.6 People’s Hospital, Qingdao, 266033, China; 2 Juan Zhao, Department of Dermatology, Qingdao No.6 People’s Hospital, Qingdao, 266033, China; 3 Jie Sun, Department of Orthopaedics, Zhangqiu Hospital of Traditional Chinese Medicine, Jinan, 250200, China; 4 Yizong Liu, Department of Orthopaedics, Qingdao No.6 People’s Hospital, Qingdao, 266033, China

**Keywords:** Fracture, Human immunodeficiency virus (HIV), Postoperative infection, Risk factors

## Abstract

**Objective:**

To evaluate the incidence of postoperative infection and fracture nonunion as well as the risk factors for postoperative infection in human immunodeficiency virus (HIV) positive patients.

**Methods:**

From May 2013 to March 2016, the HIV positive fracture patients treated surgically in orthopaedics department of our hospital were analyzed retrospectively, and fifty HIV negative fracture patients during the same period were selected as control. The clinical data of included patients were reviewed. The incidence of postoperative infection and fracture nonunion were compared between the two groups, and the risk factors for postoperative infection in HIV positive patients were evaluated.

**Results:**

The incidence of poor wound healing and incision infection in HIV positive group was higher than that in HIV negative group, but there were no significant differences between the two groups (p>0.05). Multivariable regression analysis demonstrated that HIV clinical category (p<0.05), CD4+T-lymphocyte category (p<0.01) and open fracture (p<0.05) were independent risk factors for postoperative wound infections, but age, gender, operation time, incision type, emergency operation, albumin and lymphocyte count were not (p>0.05). There was no significant difference in the rate of nonunion between the two groups (p>0.05).

**Conclusion:**

The incision can be healed, and fracture can be united normally in most of HIV positive patients with fracture, and postoperative wound infections were significantly associated with HIV clinical category, CD4+T-lymphocyte category and open fracture.

## INTRODUCTION

Acquired immune deficiency syndrome (AIDS), arising from human immunodeficiency virus (HIV) infection, is one of the largest pandemics of the modern age.^1^,^2^ In China, there were 4.34 hundred thousand persons infected with HIV, and the newly diagnosed cases were 0.7 hundred thousand in 2013.^3^ HIV-positive subjects may encounter opportunistic infections and malignancies, because of cellular immunological, humoral and non-specific deficiencies.^4^,^5^ At the same time, highly active antiretroviral therapy, which has been widely used and transformed HIV infection to a chronic, manageable condition, results in the increase of life expectancy of HIV/AIDS patients as well as the number of HIV-positive patients undergoing orthopaedic surgeries.^1^

Many clinical studies have been published to report the clinical outcomes of orthopaedic surgeries in HIV-positive patients. Some authors suggest that HIV-positive patients have higher rates of wound infection and nonunion than HIV negative patients after internal fixation.^1^,^6^ However, in a study of 42 HIV-positive patients, Hao^7^ suggests that HIV infection does not correlate with a higher rate of postoperative infection and fracture nonunion. Li also advocates that the wound in HIV positive patients can be normally healed and no infection occur in most of patients.^3^ In addition, some authors suggest that implant sepsis may occur after internal fixation, demonstrating that removal of implants in HIV-positive individuals should be considered,^5^,^8^ but in another study of 91 HIV-positive patients, Graham concluded that it was safe to perform internal fixation and no increased risk of implant sepsis was detected.^9^ Apparently, controversies are common.

Moreover, in terms of the risk factors correlated with postoperative infection in HIV positive patients, Abalo suggested that they were associated with HIV clinical category B, CD4+ T-lymphocyte category of ≥2, and contaminated wounds.^1^ Li advocated that multi-factors including age, constitutional index, operation time, wound contamination and emergency operation were correlated to the postoperative infection.^3^ Up to now, there are different viewpoints in this regard. It is critical to carry out a study to clarify these issues.

Therefore, we retrospectively reviewed the HIV positive patients with fractures treated surgically in our hospital from May 2013 to March 2016. The purpose of our study was to evaluate: (1) the incidence of postoperative infection and fracture nonunion, and (2) the risk factors for postoperative infection in HIV positive patients, to help surgeons make better treatment strategies for these patients.

## METHODS

From May 2013 to March 2016, sixty-nine fracture patients with HIV positivity who had been treated surgically in orthopaedics department of our hospital were analyzed retrospectively. The clinical data including age, gender, date of admission, operation time, albumin, hemoglobin, lymphocyte count, operation time, incision type, CD4+T-lymphocyte count, HIV clinical category, postoperative comorbidities, emergency operation or not, wound class, fracture type, surgery type, surgical wound infections, follow-up and outcomes were reviewed.

The inclusion criteria were: (1) patients with proven HIV positive who underwent operation for fracture internal fixation; (2) integrated clinical records; (3) at least 15 months of follow-up. The HIV status of patients was confirmed by enzyme-linked immunosorbant assay for HIV antibody and by western blot.^1^ Our exclusion criteria were: (1) patients with incomplete clinical data; (2) patients with difficult to heal fractures such as femoral neck fracture, talus bone fracture, scaphoid bone fracture, and so on; (3) those with preexisting infection before surgery; (4) patients with diabetes, tuberculosis, malignant tumor or hepatic-nephrotic disease, because these diseases may affect the wound healing. In addition, to facilitate the study, only patients with single fracture were included and multiple fractures were excluded.

At the same time, fifty HIV negative patients with fracture during the same period were selected as control, whose age, gender, fracture site, surgery type are similar as those in HIV positive group. The study was approved by the institutional review board of our hospital. All patients provided informed written consents at the beginning of the study.

To evaluate the immune system status of HIV positive patients, CD4+ T cell count was measured and classified into three levels, < 200/μL, 200-499/μL, and ≥ 500/μL. A CD4+ T cell count of < 200/uL indicates a high risk of opportunistic infection.^10^ In addition, postoperative infection was diagnosed according to the criteria of Center for Disease Control/National Healthcare Safety Network.^7^ Fracture union was considered when there was both full pain-free weight bearing and established bridging callus across at least two cortices on anteroposterior and lateral radiographs.^6^

The statistical analysis was carried out using SPSS 21.0 (SPSS Inc., Chicago, IL, United States). The continuous variables were presented as mean ± SD and compared by Analysis of variance. The categorical variables were compared by chi-squared test. Multivariate regression analysis was used to identify independent risk factors associated with postoperative infection. A *P* value < 0.05 was regarded as statistical significance.

## RESULTS

In this study, fifteen patients were excluded because of failing to meet the inclusion criteria, and finally fifty-four patients were included in HIV positive group. Of the 54 patients, 42 (77.8%) were males and 12 (22.2%) were females, and the mean age was 39.6 years, ranging from 23 to 72 years. In the control group of 50 HIV negative patients, 36 (72%) were males and 14 (28%) were females, and the mean age was 41.5 years, ranging from 20 to 69 years. All the patients were treated surgically with internal fixation. The baseline characteristics of two groups are shown in Table-I, there was no significant difference in age, gender, fracture site and surgery type between the two groups (p>0.05). However, in terms of CD4+ T cell count, it was significantly lower in HIV positive group than that in the control group (p<0.05).

**Table-I:** The baseline characteristics of patients in the two groups.

*Variables*	*HIV positive*	*HIV negative*	*P-value*
Number	54	50	-
Gender(M/F)	42/12	36/14	P>0.05
Age (year)	39.6±7.8	41.5±6.3	P>0.05
***Fracture site***			
Upper limb	18	13	P>0.05
Lower limb	22	19	
Spine	8	10	
Pelvis	6	8	
Surgery type			P>0.05
Internal fixation of plate	29	21	
Internal fixation of medullary pin	17	19	
Inter body fusion +internal fixation	8	10	
CD4+ T cell count	379±69	685±97	P<0.05

In 54 HIV positive patients, incision had healed normally in forty-one cases, and poor healing was found in 13 patients, including incision redness and swelling in four cases, wound discharge in two cases, fat liquefaction in one case, wound dehiscence in two cases and incision infection in four cases, the incidence of poor incision healing and infection was 24.1% and 7.4% respectively. In HIV negative group, incision was healed normally in 42 cases and poor healing was found in 8 cases, including incision redness and swelling in two cases, wound discharge in two cases, incision hematoma in one case, wound dehiscence in one case and incision infection in two cases, the incidence of poor incision healing and infection was 16% and 4% respectively. The incidence of poor healing and incision infection in HIV positive group was higher than that in HIV negative group, but there were no significant differences between two groups (p>0.05). All the infections resolved after debridement, dressing change and antibiotic treatment, and no infections developed chronic osteomyelitis.

**Fig.1:**
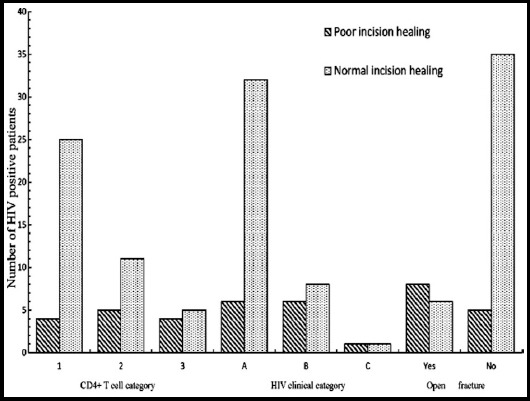
The comparison of CD4+ T cell category, HIV clinical category and open fracture or not in the two HIV positive subgroups.

The 54 HIV positive patients were divided into normal and poor incision healing subgroups. The comparison of age, albumin, hemoglobin, lymphocyte count, operation time, incision type, CD4+T-lymphocyte category, HIV clinical category, open fracture and emergency operation between two subgroups is listed in Table-II, and there were significant differences in above variables between two subgroups (p<0.05, Table-II, Fig.1). However, multivariable egression analysis demonstrated that HIV clinical category (p<0.05), CD4+T-lymphocytecategory (p<0.01) and open fracture(p<0.05) were independent risk factors for postoperative wound infections, but age, gender, operation time, incision type, emergency operation, albumin and lymphocyte count were not (p>0.05).

**Table-II:** The comparison between the two HIV positive subgroups.

*Variables*	*Poor incision healing*	*Normal incision healing*	*P-value*
Age (year)	45.1±6.7	38.6±4.8	P<0.05
Albumin(g/L)	29.8±4.6	34.9±6.3	P<0.05
Hemoglobin(g/L)	125.6±16.4	136.5±17.9	P<0.05
Lymphocyte count(x10^9^/L)	1.2±0.5	1.6±0.8	P<0.05
Operation time(min)	182±36	140±25	P<0.05
***Incision type (n)***			
I	2	33	P<0.05
II	3	6	
III	8	2	
***CD4+ T cell category(n)***			
1	4	25	P<0.05
2	5	11	
3	4	5	
***HIV clinical category(n)***			P<0.05
A	6	32	
B	6	8	
C	1	1	
***Emergency operation (n)***			
Yes	9	13	P<0.05
No	4	28	
***Open fracture***			P<0.05
Yes	8	6	
No	5	35	

Postoperatively, in HIV positive group, three fractures including one tibia and two humerus fractures failed to unite, which underwent revision surgery resulting in a final union. The nonunion rate in HIV positive group was 5.6%. In the control group, one humerus fracture failed to unite, the rate of nonunion was 2%. There was no significant difference in the rate of nonunion between the two groups (p>0.05).

## DISCUSSION

In this study, we focused on the evaluation of the incidence of postoperative infection and fracture nonunion as well as the risk factors for postoperative infection in fracture patients with HIV positivity, to clarify the controversial viewpoints, help surgeons better understand the issues.

We found the incidence of incision infection in HIV positive group was higher, but there was no significant difference between the two groups, demonstrating that HIV positivity does not significantly increase the incidence of postoperative infection. The result is consistent with Li’ study.^3^ At the same time, the incidence of poor incision healing in HIV positive patients was 24.1%, which is far higher than the value of 16% in HIV negative patients, but there was no significantly difference between the two groups. The result was different from Li’ study. In our opinion, it is attributed to the small sample size in this study, and a large scaled study may be needed to clarify the issues in the future.

In addition, we found that in the HIV positive group the value of albumin, hemoglobin, lymphocyte count in patients with poor incision healing was lower than those with normal incision healing, indicating that nutrition support plays an important role in the normal healing of patients. (Table-II) Many studies have advocated the same viewpoints, suggesting reasonable perioperative adjuvant treatment, including nutritional support, immunomodulators and anti-retroviral drugs, should be highly recommended.^11^,^12^ In the current study, most patients with normal incision healing were offered with nutrition support during perioperative periods, which also support the above viewpoints.

Some scholars studied the risk factors related to postoperative wound infection in HIV positive patients^1^,^3^. In the current multivariable regression analysis, we found that postoperative wound infections were significantly associated with HIV clinical category, CD4+ T-lymphocyte category and open fracture, which was consistent with Abalo’s conclusion, because the wounds may be contaminated in most open fractures. Some authors also hold the same views. In a review of twenty-six patients, Wijesekera and colleagues suggested in open fractures managed with internal fixation, postoperative wound infection rates were significantly increased in HIV-positive population compared to HIV-negative controls.^13^

In terms of fracture union, some studies suggested that HIV infection and its treatment have been linked with an altered bone mineral density and cytokine environment as well as increased avascular necrosis, which results in increased risks of fracture nonunion.^14^ In the current study, the nonunion rate in HIV positive group was 5.6% and in control group the rate was 2%, although the rate in HIV positive group was higher, no significant difference was found between the two groups. Different from above viewpoints, we found the HIV positivity was not associated with high risk of fracture nonunion.

### Limitations of the study.

First, the current study was a retrospective instead of a prospective one, although a randomized controlled trial is difficult to carry out in this regard, a prospective study may be better in explaining the current issues. Second, the sample size of this study was small, and some significant results can’t be achieved, which may affect the readers to understand correctly some controversial viewpoints in this field. While, despite limitations, we can conclude from this study that the incisions can be healed, and fracture can be united normally in most of HIV positive patients with fracture, and postoperative wound infections were significantly associated with HIV clinical category, CD4+T-lymphocyte category and open fracture.

### Authors’ Contribution

**GYX, JZ** conceived, designed & editing of manuscript. **JS** did statistical analysis.

**GYX, YZL, JZ** did data collection, manuscript writing and performed review and final approval of manuscript.
